# Enhancing the properties of high-density oil well cement with Qusaiba kaolinite

**DOI:** 10.1038/s41598-024-76914-9

**Published:** 2024-10-29

**Authors:** Abdulmalek Ahmed, Ahmed Abdulhamid Mahmoud, Salaheldin Elkatatny, Dhafer Al Shehri, Korhan Ayranci

**Affiliations:** 1 Department of Petroleum Engineering, King Fahd University of Petroleum and Minerals, 31261 Dhahran, Saudi Arabia; 2 Department of Geosciences, College of Petroleum Engineering and Geosciences, King Fahd University of Petroleum and Minerals, 31261 Dhahran, Saudi Arabia

**Keywords:** Oil well cementing, Qusaiba Kaolinite, Rheology, Thickening time, Chemical engineering, Civil engineering

## Abstract

High-density cement slurries used in oil well cementing often face challenges such as particle settling, poor rheological properties, permeability, and compressive strength degradation, which can compromise zonal isolation and well integrity. This study focuses on using kaolinite, a clay mineral, as an additive due to its potential to improve the performance of high-density cement by modifying key properties. Several concentrations of kaolinite were examined to evaluate their influence on several cement properties such as rheology, thickening time, permeability, porosity, and compressive strength. Additionally, it assesses the impact of kaolinite on cement sheath solids settling using both conventional methods and nuclear magnetic resonance (NMR). The results revealed that an optimal concentration of 1% kaolinite by weight of cement (BWOC) significantly reduced particle settling by 74.4%, enhanced compressive strength by 13%, and lowered permeability and porosity by 74% and 7%, respectively. Additionally, kaolinite improved rheological properties by an 8.4% reduction in plastic viscosity, a 19.4% increase in yield point, and a 30% increase in gel strength. Kaolinite also acted as a retarder, increasing thickening time. These improvements contribute to better cement sheath integrity and wellbore stability, highlighting kaolinite’s potential as an effective additive for high-density cement.

## Introduction

Cementing is an important process in oil well construction and maintenance, essential for well stability throughout its lifecycle. This process is vital for preventing mud contamination with formation fluids by ensuring effective zonal isolation throughout the drilling and cementing processes^1^. Cementing fills the annular space between the casing and the wellbore wall^2,3^, creating a seal that prevents formation fluids from entering the wellbore and mixing with the drilling mud. This barrier isolates different geological zones and blocks the pathways for fluid migration.

Cementing’s importance extends beyond structural support, playing a key role in maintaining well integrity and addressing various operational issues^4,5^. It serves as a remedial measure for casing defects caused by leaks^6^ or corrosion^7^, effectively sealing off problematic sections and extending the well’s productive lifespan. In deep well environments, the cement sheath acts as a shock absorber^8^, dissipating energy from dynamic loads, and mitigating the risk of well failure and catastrophic blowouts^9^.

Successful cementing requires careful consideration of factors such as formation sections, borehole conditions, and well depth. Achieving a strong bond between cement and formation demands meticulous planning of cement properties^10^, including density, viscosity, setting time, permeability, and strength development, to meet each well’s specific requirements. In high-pressure, high-temperature (HPHT) environments, conventional cement formulations often fall short. These extreme conditions demand specialized high-density oil well cement, engineered to withstand intense pressures and temperatures encountered in deep formations^11^.

The high-density cement is suitable for deep wells with high pressures and temperatures due to its increased flexibility compared to regular cement^12^, allowing it to maintain integrity under conditions that would compromise standard formulations. However, the application of these slurries comes with technical challenges. One of the main issues is ensuring the proper balance between slurry density and its other properties such as viscosity, setting time, permeability, and strength development. If the cement slurry is too dense, it can lead to excessive pressure on the wellbore, potentially causing fracturing or fluid losses.

The development of heavy-weight cements relies heavily on incorporating weighting agents to increase the overall density of the cement slurry. The most widely used weighting agents are barite, hematite, and ilmenite^13^. Each of these materials brings unique properties to the cement mixture, allowing engineers to fine-tune cement characteristics to match specific well requirements. The most commonly used weighting material to increase the density of Class G cement is hematite. Class G oil well cement is a specialized type of Portland cement designed for use in oil and gas well construction. It is primarily composed of tricalcium silicate (C_3_S) and dicalcium silicate (C_2_S), which are responsible for early and long-term strength development, respectively. It has a low tricalcium aluminate (C_3_A) content, which enhances sulfate resistance and prevents undesirable fast setting in oil well environments. Tetracalcium aluminoferrite (C_4_AF) is present in smaller amounts, contributing to color and process efficiency. A small amount of gypsum is added to regulate the setting time. The composition of Class G cement is tailored to withstand high temperatures and pressures encountered in deep well environments^2,14^.

In recent years, the oil and gas industry has shown increasing interest in exploring alternative additives that could enhance cement performance while potentially offering economic or environmental benefits. Kaolinite, with its unique structure and composition, presents intriguing possibilities for oil well cementing applications. Kaolinite, a clay mineral, has a structure composed of a single silica tetrahedron sheet combined with aluminum in an octahedral shape^15^. It’s a white chemical consisting of silicate (SiO_2_), aluminium oxide (Al_2_O_3_), and water (H_2_O) in proportions of 46.54%, 39.5%, and 13.96%, respectively^16^. Compared to other clays like illite, kaolinite is non-swellable^17^.

In the oil industry, kaolinite is primarily used in drilling mud. Adebayo and Ajayi showed that increasing kaolin percentage in mud increases the fluid density^18^. Adogbo and Mohammed used kaolin hydrated with starch, resulting in higher density compared to barite^19^. Adeboye and Oyekunle explored using kaolinite as a bentonite substitute, finding it decreases viscosity and increases mud density^20^. Amer et al. used kaolin (25 − 50%) instead of bentonite in water-based mud, increasing densities and decreasing viscosities and yield points^21^.

Several experimental studies have provided promising evidence regarding kaolinite’s ability to enhance cement properties in the concrete industry. For example, Gerasimov et al. demonstrated that adding kaolinite to Portland cement increases compressive strength by 20% . Sabir et al. highlighted the enhancement in compressive strength, reduced permeability, and improved resistance to chemical attacks when kaolinite is used as a partial replacement for Portland cement^23^. Badogiannis et al. demonstrated that the inclusion of kaolinite-based materials significantly improves the long-term durability of cement, particularly in resisting sulfate attack and reducing chloride ion permeability^24^. Rashad discussed how the use of kaolinite improves cement’s thermal stability and fire resistance due to the formation of stable hydrates^25^. Wild et al. observed that kaolinite addition led to a denser microstructure and reduced porosity in cement pastes^26^. Courard et al. reported improved resistance to chloride penetration in concrete containing kaolinite^27^. Hassannezhad et al. discussed how kaolinite enhances the strength, microstructure, and porosity of blended cement^28^. The finding that kaolinite can significantly enhance cement properties suggests it could play a valuable role in developing stronger, more resilient cement formulations for oil well applications. For instance, Adjei et al. demonstrated that a lightweight cement formulation with kaolinite as a 30% replacement for cement has excellent mechanical properties that are particularly suitable for applications within downhole oil wellbore environments^29^. Given the promising results observed in both drilling fluid and cement applications, there is growing interest in further exploring kaolinite’s potential as an additive in oil well cementing. However, there is no application of kaolinite in heavy-weight oil well cement.

Therefore, this research aims to provide comprehensive insights into how kaolinite affects various critical properties of heavy-weight oil well cement, including rheology, thickening time, particle settling, permeability, porosity, and compressive strength. By conducting thorough investigations into these aspects, this study aims to unlock the full potential of kaolinite as an additive for high-density cement by developing innovative cement formulations that offer improved performance, durability, and cost-effectiveness compared to traditional options. Such advancements could lead to more resilient well constructions, enhanced operational efficiency, and potentially reduced environmental impact in oil and gas operations.

## Materials and methods

### Collected materials

This research utilizes Class G cement and Qusaiba kaolinite as primary materials, supplemented by various chemical additives to enhance oil well cement performance across diverse wellbore conditions. These additives include silica flour, defoamer, dispersants, fluid loss agents, and retarder. Additionally, hematite is employed to increase cement slurry density, making it suitable for high-pressure, high-temperature applications. The used hematite has a high specific gravity of 4.95. The Qusaiba kaolinite, sourced as a raw material from Saudi Arabia, is combined with Class G cement and other additives obtained from a local service company. The cement slurries are prepared using deionized water.

**Fig. 1:**
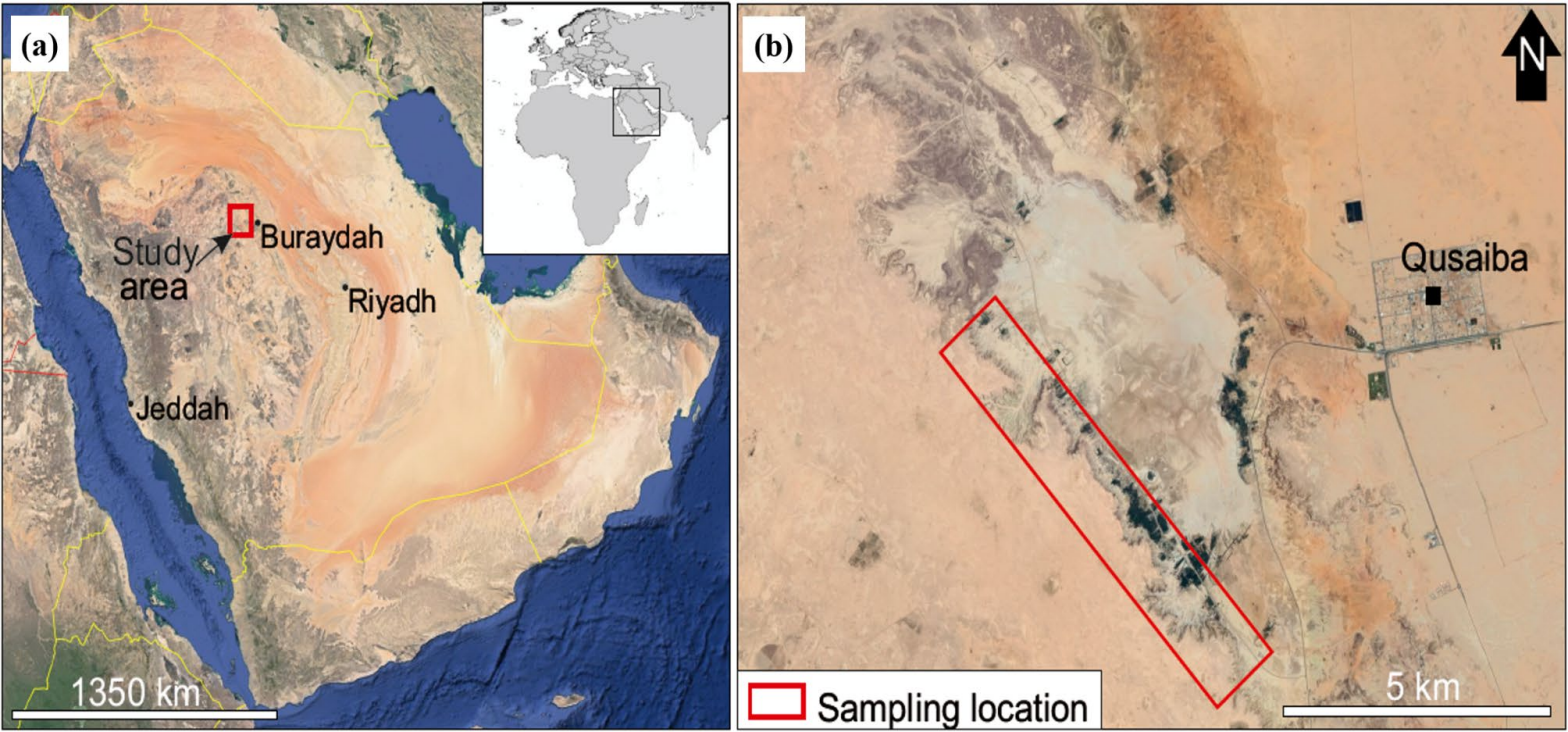
Geographical context of Saudi kaolinite: (**a**) regional map highlighting the study area, and (**b**) specific location of kaolinite sampling within the Qassim Region^30^.

The Qusaiba kaolinite originates from the Silurian-age Qusaiba Member of the Qalibah Formation, located in the Qassim Region of central Saudi Arabia as shown in Fig. 1. This geological formation has an exposed thickness of approximately 30 m and is characterized by gray to green mudstones. Rare mica-rich sandstone alternations are observed towards the upper section. The Qusaiba Member is conformably overlain by the sand-rich Sharawra Member of the same formation, and in some areas, it is erosively overlain by the Tawil Formation. It’s worth noting that the base of the Qusaiba Member, sometimes informally referred to as the “hot shale,” is not exposed in the Qassim Region.

### Material characterization

**Fig. 2:**
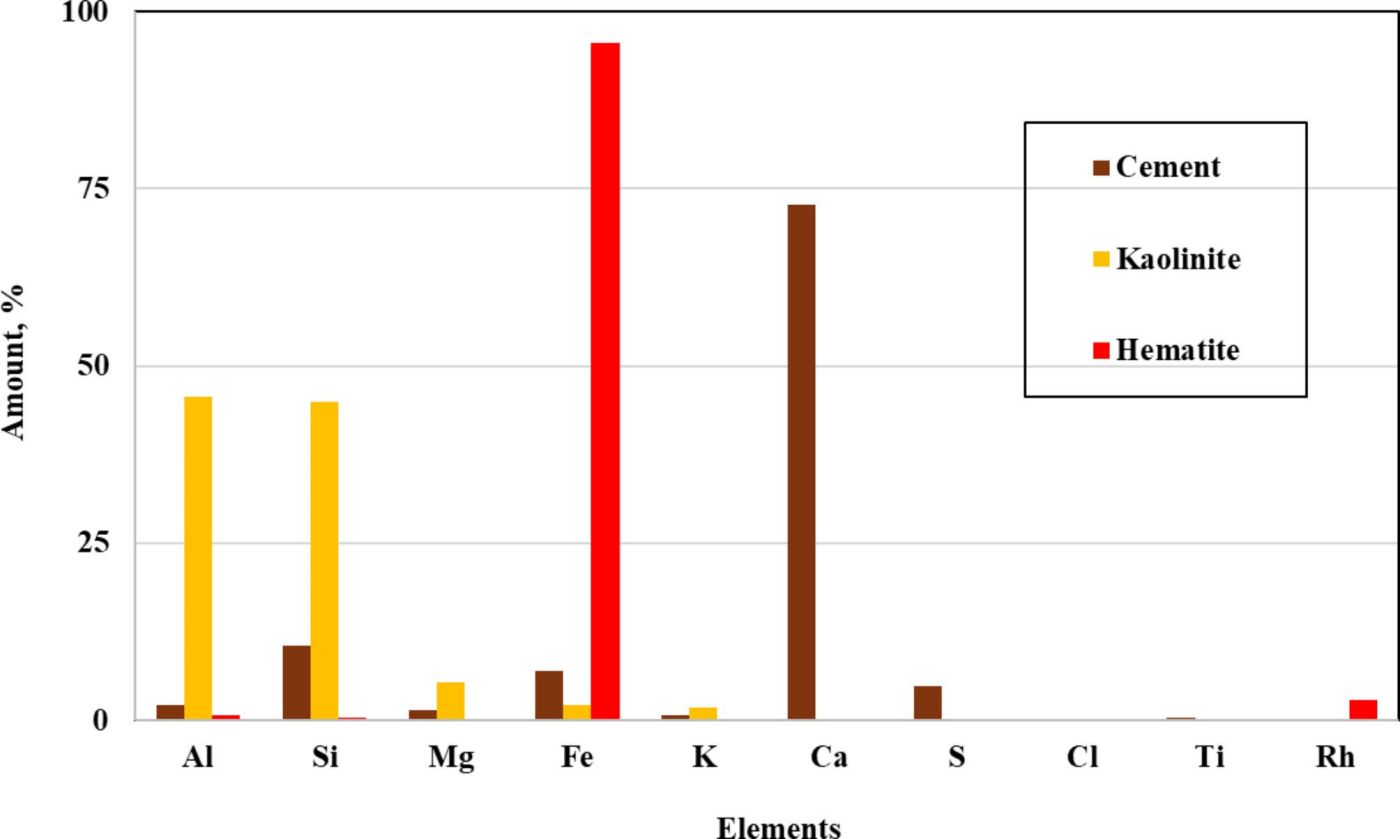
Elemental composition (in percentage) of the cement, Qusaiba kaolinite, and hematite as determined by XRF.

The elemental composition of Class G cement, Qusaiba kaolinite, and hematite was analyzed using X-ray Fluorescence (XRF). Figure 2 presents the primary elements found in each material. Class G cement is predominantly composed of calcium (Ca = 73%), followed by silicon (Si = 11%) and iron (Fe = 7%). Qusaiba kaolinite primarily contains aluminium (Al = 47%) and silicon (Si = 45%), while hematite is almost entirely iron (Fe = 97%).

The high calcium content in the cement, combined with the aluminium and silicon in kaolinite, is expected to trigger a pozzolanic reaction. During the hydration process, the rapid hydration of tricalcium silicate and tricalcium aluminate, as well as the slower hydration of tetracalcium ferroaluminate and dicalcium silicate, leads to the formation of calcium silicate hydrates (CSH). This reaction between silicon and calcium ions contributes to the enhancement of the cement’s strength^31,32^. Additionally, aluminium integrates freely into the cement’s CSH structure, significantly influencing various aspects of the cement’s chemical performance^33–36^.

Particle size distribution analysis was conducted using the Nano tec plus instrument with a wet dispersion unit. The results, illustrated in Table 1, reveal that Qusaiba kaolinite has a notably small average particle size (D_50_) of less than 3.6 μm, indicating very fine particles. In comparison, cement and hematite have larger D_50_ values of 12.2 μm and 9.4 μm, respectively. The fine nature of the kaolinite particles suggests potential benefits in reducing the cement specimens’ petrophysical properties and particle settling by filling pore spaces and leading to denser and stronger cement.

**Table 1:** The particle size distribution of Class G cement, Qusaiba Kaolinite, and hematite.

Material	Cement	Kaolinite	Hematite
D_10_	2.7	0.95	3.1
D_30_	9.8	2.03	5.9
D_50_	12.2	3.68	9.4
D_70_	14.7	6.8	12.6
D_90_	18.7	14.3	17.2

**Fig. 3:**
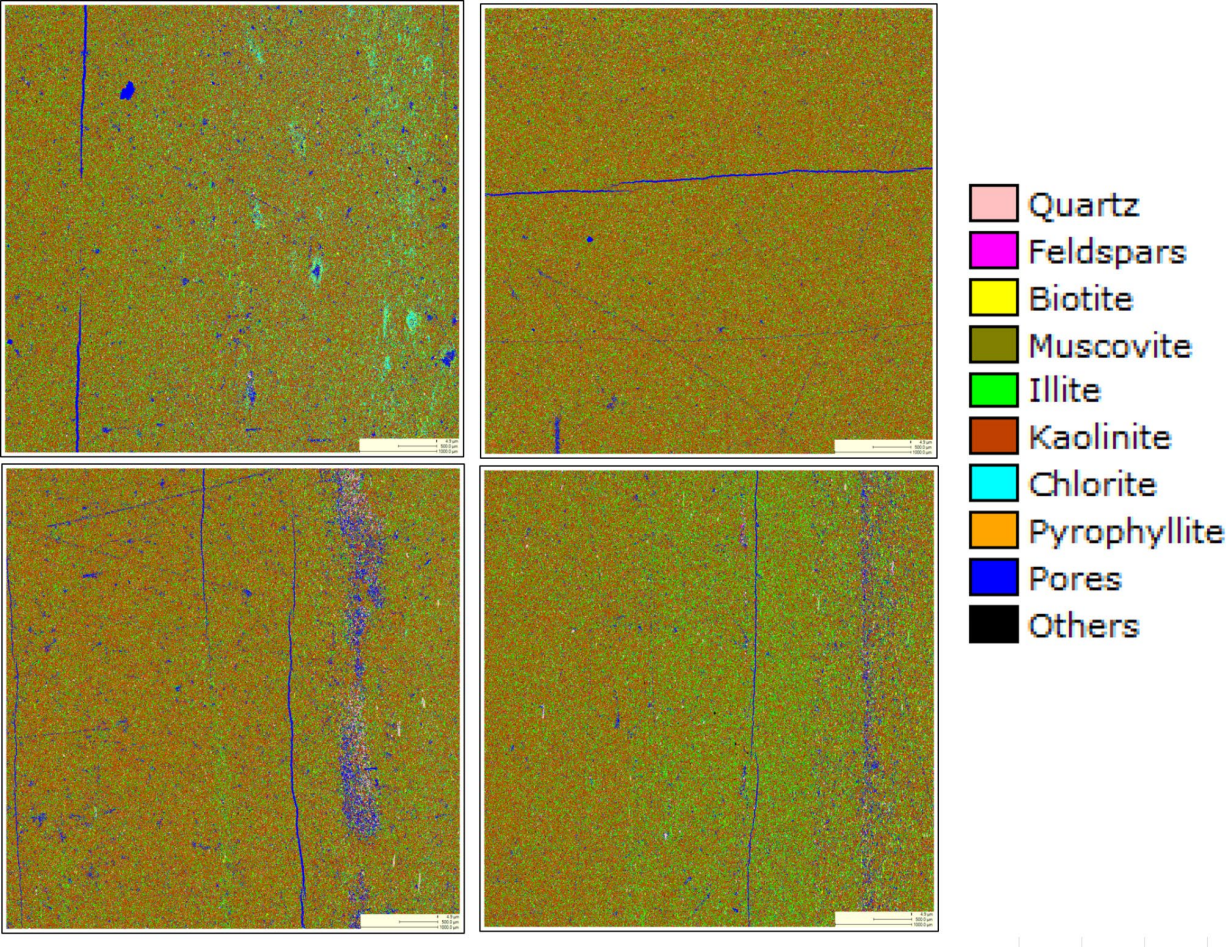
Results of QEMScan for kaolinite.

To further characterize the material composition, FEI QEMscan 650 F (quantitative evaluation of materials scanning) was employed, with results presented in Fig. 3. This analysis indicates that approximately 61% of the rock samples obtained consist of kaolinite, while illite and quartz comprise around 27% and 6%, respectively.

### Slurries preparation

Six cement slurries were prepared according to API standard 10- A^14^. Table 2 summarizes the composition of these cement mixtures. The primary variable among the samples is the percentage of kaolinite, while all other components remain constant across the mixtures.

Six cement slurries were prepared with kaolinite concentrations ranging from 0 to 4% by weight of cement (BWOC), as detailed in Table 2. The base sample contains no kaolinite, followed by samples with 0.25%, 0.5%, 1%, 2%, and 4% BWOC kaolinite, respectively. All slurries incorporated hematite to achieve a high-density cement slurry of 18 lb/gal.

The preparation process began with the dry mixing of the cement, silica flour, and hematite, followed by wet mixing of the other components in Table 2 and the addition of the dry mix. The blending process involved high-speed mixing with subsequent incorporation of components. Following this, the cement slurries were transferred to a digital atmospheric consistometer for conditioning at 194 °F for 30 min. After this process, the samples were ready for preparation into the required dimensions for testing.

**Table 2:** Formulations of cementing mixtures, (%BWOC).

Components	Base Cement	Sample 1	Sample 2	Sample 3	Sample 4	Sample 5
Cement	100	100	100	100	100	100
Silica Flour	35	35	35	35	35	35
Hematite	32.9	32.9	32.9	32.9	32.9	32.9
Water	44	44	44	44	44	44
Defoamer	5 × 10^−3^	5 × 10^−3^	5 × 10^−3^	5 × 10^−3^	5 × 10^−3^	5 × 10^−3^
Fluid Loss	0.5	0.5	0.5	0.5	0.5	0.5
Dispersant	0.25	0.25	0.25	0.25	0.25	0.25
Retarder	0.5	0.5	0.5	0.5	0.5	0.5
Kaolinite	0	0.25	0.50	1.00	2.00	4.00

### Experimental program

This study evaluated a comprehensive range of cement sample properties, including rheology, thickening time, cement settling, nuclear magnetic resonance (NMR) characteristics, porosity, permeability, and compressive strength. Following the initial conditioning phase, the cement slurries underwent specific testing procedures tailored to each property of interest.

For rheological assessment, the conditioned slurries were transferred to a viscometer, which provided detailed measurements of the cement’s flow behavior under various shear conditions. Also, a high-pressure high-temperature (HPHT) consistometer was employed to determine the thickening time of the cement slurry, a critical parameter for ensuring adequate pumping time during well cementing operations.

To assess the additional properties, it was essential to work with hardened cement specimens. The process involved shaping the prepared mixtures into two different forms: cube-shaped samples with sides of 2 inches, and cylindrical samples measuring 4 inches in height and 1.5 inches across. Then, these specimens underwent a hardening process within a specialized high-pressure, high-temperature (HPHT) environment. The samples were exposed to precisely regulated conditions, with the curing chamber maintained at 294 °F and 3000 psi for a full day. This curing regime ensured the cement samples achieved the desired set and strength characteristics, closely simulating downhole conditions and preparing the specimens for subsequent testing of solid-state properties such as particle settling, porosity, permeability, and compressive strength.

The conditions for thickening time and curing were selected based on an actual field formulation for heavyweight oil well cement used in drilling deep high-pressure high-temperature (HPHT) formations. For this well, the bottom hole circulating temperature (BHCT) was 220 °F, the bottom hole static temperature (BHST) was 294 °F, and the bottom hole pressure (BHP) was 12,300 psi. For the thickening time test, the conditions simulate the material’s behavior during placement. Therefore, a temperature of 220 °F was chosen to replicate the actual BHCT, and a pressure of 12,300 psi was used to reflect the actual BHP. The HPHT consistometer used has an upper-pressure limit of 50,000 psi. For the curing process, the conditions simulate the long-term environmental exposure. Therefore, a temperature of 294 °F was selected to replicate the BHST, while a pressure of 3,000 psi was chosen due to the curing chamber’s upper-pressure limit of 5,000 psi. Additionally, according to API Standard 10-A^14^, the maximum required pressure for curing is 3,000 psi. This pressure level allows for testing at elevated conditions while maintaining a safety margin below the equipment’s maximum capacity. Lastly, the duration of 24-hour curing time follows the API standard for curing periods of 24 h, ensuring consistency with industry practices.

It should be mentioned that the reported values in the manuscript are the average of three readings. Where every measurement was repeated three times, and the average was taken. So, three samples were prepared for each test.

#### Evaluation of rheological properties

To accurately assess the rheological properties, a systematic approach was employed in this study. Following the conditioning phase, each cement slurry was carefully transferred into a viscometer for analysis. The viscometer was calibrated and set to operate at 194 °F, simulating relevant downhole conditions. This instrument was used to measure several key rheological parameters such as plastic viscosity, yield point, 10-second, and 10-minute gel strength.

The evaluation process involved measuring shear stress at various shear rates, corresponding to rotational speeds of 3, 6, 100, 200, and 300 rpm. To ensure accuracy and account for potential thixotropic behavior, measurements were taken in both ascending and descending order of rotation speeds. The final values for each parameter were then calculated as the average of these bidirectional measurements.

This rheological assessment provides valuable insights into the cement slurry’s flow behavior, which is essential for predicting pumping requirements, optimizing placement strategies, and ensuring successful cement jobs in challenging wellbore environments.

#### Evaluation of thickening time

The thickening time of the cement slurry was determined using a high-pressure, high-temperature (HPHT) consistometer, simulating downhole conditions with precision. This critical test was conducted under rigorous parameters, specifically at a pressure of 12,300 psi and a temperature of 220 °F.

In this context, the thickening time is defined as the duration required for the cement slurry to reach a consistency of 70 Bearden units (Bc). The consistency of 70 BC is frequently utilized as a parameter to estimate the timeframe during which the slurry will retain its fluid characteristics. This threshold is significant because it represents the point at which the cement slurry becomes too viscous to be effectively pumped, marking the practical limit of its workability. On the other hand, the duration required for the slurry to achieve a measurement of 100 Bc is regarded as the maximum threshold. In which the slurry undergoes solidification, rendering pumping unfeasible. Therefore, in practice, the desired thickening duration is frequently determined by the specific goals of the operator. Typically observed consistencies include 40, 50, 60, and 70 Bc^29,37,38^.

The consistometer continuously monitors the cement slurry’s consistency over time, providing a comprehensive profile of its thickening behavior. This measurement is crucial for well engineers, as it informs decisions regarding pumping schedules, additive adjustments, and overall cementing operation planning. By accurately determining the thickening time under these specific HPHT conditions, operators can ensure that the cement remains pumpable for the duration required to complete the cementing process, thereby minimizing the risk of premature setting and potential well-completion issues.

#### Evaluation of cement settling

Density variation along the cement column is a significant concern in heavy-weight cement applications, with the bottom section often becoming denser than the top due to the sedimentation of hematite^39^. To thoroughly investigate this phenomenon, the present study employed two distinct techniques: a conventional sedimentation test and nuclear magnetic resonance (NMR) analysis.

- Conventional sedimentation test

This method involved a systematic approach to quantify density variations within the cement samples. Each sample was precisely sectioned into three equal parts, representing the bottom, middle, and top of the cement column. To ensure a fair comparison, all sections were standardized to dimensions of 1.5 inches in diameter and 0.5 inches in length. The mass-to-volume relationship was used to determine the density of individual segments. To evaluate the extent of density variation, a comparative analysis was performed. This involved calculating the disparity between the uppermost and lowermost segment densities and then expressing this difference as a fraction of the bottom segment’s density. This approach provided a quantitative measure of density stratification within the sample.

b. Nuclear magnetic resonance (NMR)

An in-depth exploration of the correlation between void space dimensions and porosity fluctuations was conducted using advanced nuclear magnetic resonance technology (NMR). Each rod-shaped cement specimen was subjected to examination via a low-intensity 2 MHz nuclear magnetic resonance spectroscope. This method facilitated a comprehensive investigation of the internal void network within the solidified cement.

The analysis focused on measuring T_2_ decay periods, quantified in thousandths of a second. These values correspond to the surface expanse of cavities, their volumetric capacity, and the relaxation characteristics of constituents lining the void boundaries. Interpretation of the T_2_ data patterns allowed for extrapolation of the void size spectrum throughout the cement samples. This approach yielded crucial information regarding the degree and mechanics of particulate stratification within the material.

#### Evaluation of petrophysical properties

The permeability and porosity of the cement matrix are crucial petrophysical properties that significantly influence the performance and integrity of well completions. To assess these properties, cylindrical samples measuring 0.7 inches in height were prepared with varying concentrations of kaolinite. These samples were precisely dimensioned to a length of 0.7 inches and a diameter of 1.5 inches to ensure consistency in measurements.

Permeability measurements were conducted using nitrogen gas under a confining pressure of 1000 psi, following the methodology described by Sanjuán and Muñoz-Martialay^40^. The permeability values were calculated based on the Hagen − Poiseuille law, which relates fluid flow through porous media to pressure differential and sample geometry.

Porosity determinations were carried out using a Boyle’s law Porosimeter, as outlined by Peters^41^. This method leverages the principle of gas expansion to accurately measure the void space within the cement matrix, providing a reliable assessment of the sample’s porosity.

By employing these standardized techniques, the study can provide a comprehensive characterization of the cement samples’ pore structure and fluid transport properties. These properties are essential for evaluating the potential impact of kaolinite addition on the cement’s ability to provide zonal isolation and resist fluid migration in wellbore environments.

#### Evaluation of the compressive strength

To evaluate the impact of kaolinite on cement strength, compressive strength tests were conducted on all prepared cement samples. This critical assessment followed the stringent guidelines set forth by the American Society for Testing and Materials^42^, ensuring reliability and reproducibility of results. The testing procedure involved subjecting cubical cement specimens to controlled, uniaxial compression using a calibrated crushing machine. This apparatus applies a gradually increasing load to the sample until failure occurs, providing a precise measurement of the maximum stress the cement can withstand before structural breakdown.

By systematically testing samples with varying kaolinite concentrations, this study can quantify the relationship between kaolinite addition and cement strength. Such measurement is invaluable for optimizing cement formulations to meet the demanding structural requirements of oil and gas well completions, particularly in high-stress environments where cement integrity is paramount for well safety and productivity.

According to API standard^14^, the minimum required compressive strength test includes curing periods of 8 h and 24 h. In addition, one-day curing is often used as an early indicator of compressive strength to assess the initial set and early strength development of cement. Therefore, one-day curing is sufficient to represent the strength characteristics accurately. However, compressive strength increases over time, and this long duration might provide a complete or representative view of the long-term strength characteristics of the cement. This long time interval allows for a more comprehensive understanding of the cement’s behavior across different curing periods.

## Results and discussion

This section discusses the influence of adding kaolinite to the properties of oil well cement in terms of rheology, thickening time, cement settling, NMR, porosity, permeability, and compressive strength.

### Results of rheology

**Fig. 4:**
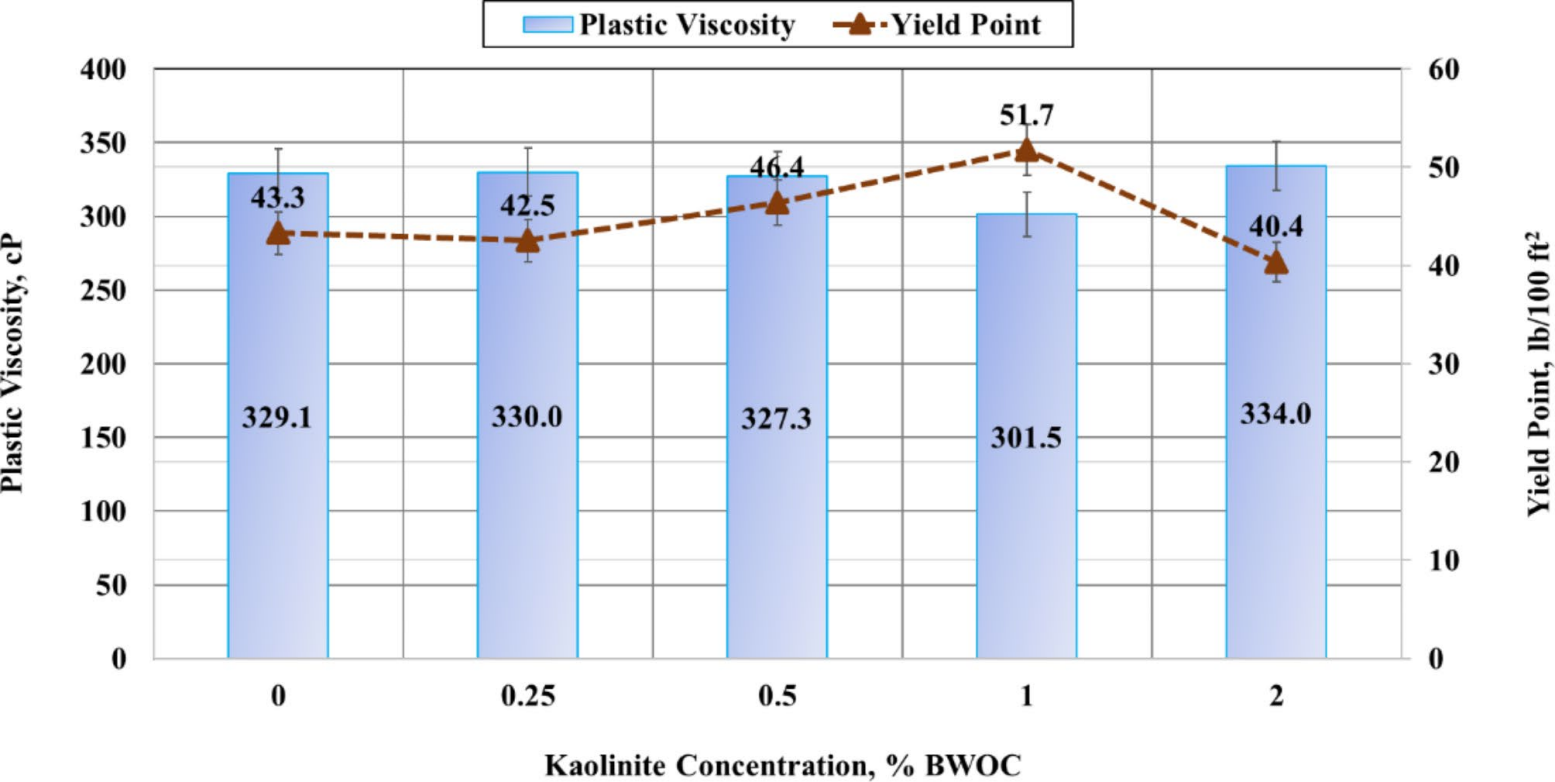
Results of viscosity and yield point.

**Fig. 5:**
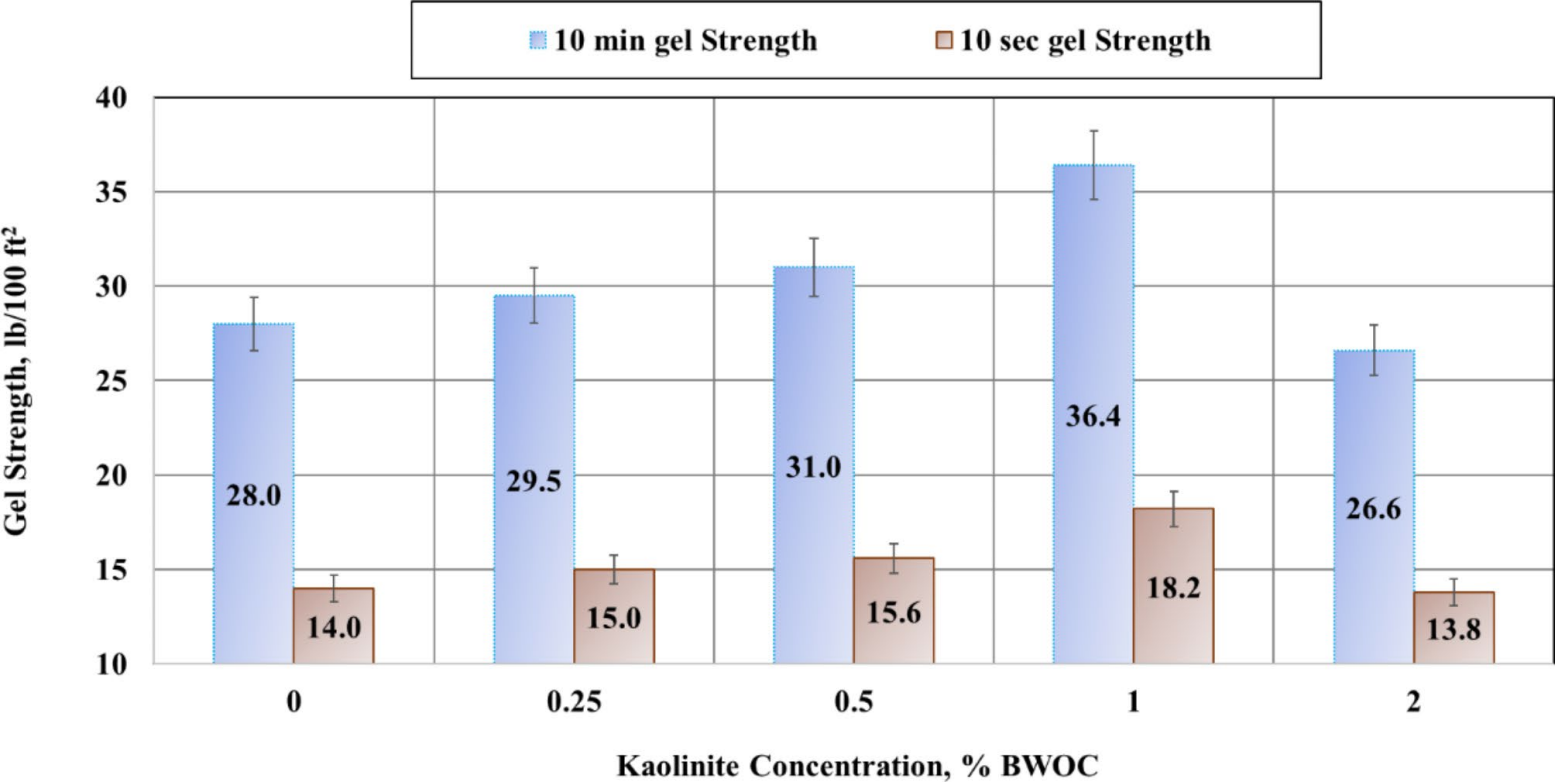
Results of gel strength.

The rheological properties of oil well cement slurries are crucial indicators of their quality and physical characteristics throughout the cementing process, from initial mixing to final setting^43^. These properties play a fundamental role in determining the cement slurry’s composition, performance, and pumpability into the wellbore. The rheological parameters were taken from measurements of shear stress at varying shear rates. Each of these parameters has specific implications for the cementing operation. Lower values of plastic viscosity are generally preferred as they correlate with reduced pumping pressures, facilitating easier placement of the cement slurry. Higher values of yield point are desirable as they enhance the slurry’s carrying capacity, ensuring proper suspension of cement particles and additives^44^. Increased gel strength is beneficial for controlling gas migration within the cement column^45,46^. The impact of increasing kaolinite concentrations on these rheological properties is illustrated in Figs. 4 and 5. These graphs provide a visual representation of how the addition of kaolinite influences the cement slurry’s flow behavior and structural characteristics.

In Fig. 4, the plastic viscosity of the base cement sample is 329.1 cP which is very high as the sample contains weighting materials. However, the addition of kaolinite to a concentration of 1% caused a reduction in the plastic viscosity, which is practical for cement pumping. Firstly, a slight increase in the viscosity to a value of 330 cP was found when 0.25% BWOC of kaolinite was added to the slurry. Then, the plastic viscosity was reduced by 0.6% and 8.4% for cement samples containing kaolinite amounts of 0.5% and 1%, respectively. However, the 2% BWOC of kaolinite increased the plastic viscosity to a value of 334 cP, which is more than the base case having no kaolinite. This increase may be due to the agglomeration of kaolinite particles at higher concentrations and reducing their efficiency. It should be mentioned that the slurry prepared with 4% BWOC kaolinite was significantly thicker than the other slurries, making it impossible to measure its rheology and thickening time. However, the slurry was still able to cure and solidify, allowing other tests to be conducted, such as sedimentation, permeability, porosity, and compressive strength.

In addition, the cement sample of 1% kaolinite was the optimum as it provided the lowest plastic viscosity of 301.5 cP. This decrease in the viscosity could be due to many factors. For instance, the large surface area of kaolinite allows kaolinite to adsorb water and other organic molecules onto its surface, which helps to lubricate the cement mixture and reduce its viscosity. Kaolinite particles are typically plate-like in shape which can affect how particles interact in suspension, potentially creating a ball-bearing effect that reduces friction between cement particles^47^. Kaolinite particles carry a negative surface charge at most pH levels relevant to cement slurries. This can lead to electrostatic repulsion between particles, which might contribute to reduced viscosity^48^. A lower plastic viscosity will allow the cement slurry to flow more easily up through the annular space, which will help to ensure that the wellbore is properly sealed.

In Fig. 4, the results of the yield point show that it varies with the concentration of the kaolinite. At 0% BWOC, the yield point was 43.3 lb/100 ft^2^. Using 0.25% BWOC of kaolinite decreased the yield point by only 0.8 lb/100 ft^2^. The yield point then increased as the dosage of the kaolinite was increased to 0.5% and 1% BWOC, reaching values of 46.4 and 51.7 lb/100 ft^2^ with an increase of 7.2 and 19.4% compared to the base cement. However, at 2% BWOC, the yield point decreased to 40.4 lb/100 ft^2^, which is even lower than the value obtained at 0% BWOC. This decrease may be attributed to the formation of excessive agglomerates at high concentrations of the kaolinite. It can be seen that the results of the yield point confirm the plastic viscosity findings. The current research findings align with previous observations, indicating an inverse correlation between the yield point and the viscosity^49^. The results suggested that the optimal concentration of the kaolinite for increasing the yield point was 1% BWOC. The increase in yield point with increasing concentration of the kaolinite might be due to forming a more robust network of particles, which resists deformation under stress. The results of this study indicate that incorporating the kaolinite material could improve the carrying ability of the cement slurry.

Moreover, the experimental data revealed comparable patterns when examining the gel strength. The results indicated that the gel strength varied with the amount of kaolinite as presented in Fig. 5. Specifically, the gel strength increased with the addition of kaolinite up to 1% BWOC and then decreased at a concentration of 2% BWOC. At 1% BWOC, the gel strength reached its maximum value of 36.6 and 18.2 lb/100 ft^2^ for the 10-minute and 10-second gel strengths, respectively, with an increase of 30% compared to the 0% BWOC of kaolinite. The improvement in the flow properties of the cement slurry led to a more stable cement mixture by optimizing the particle suspension in the mixture while maintaining minimal viscosity. The improved yield point and gel strength of the slurry ensured that the solids were suspended even under dynamic and static conditions, emphasizing the impact of density variation, as will be discussed in Sect. Results of particle sedimentation through conventional method.

### Results of thickening time

**Fig. 6:**
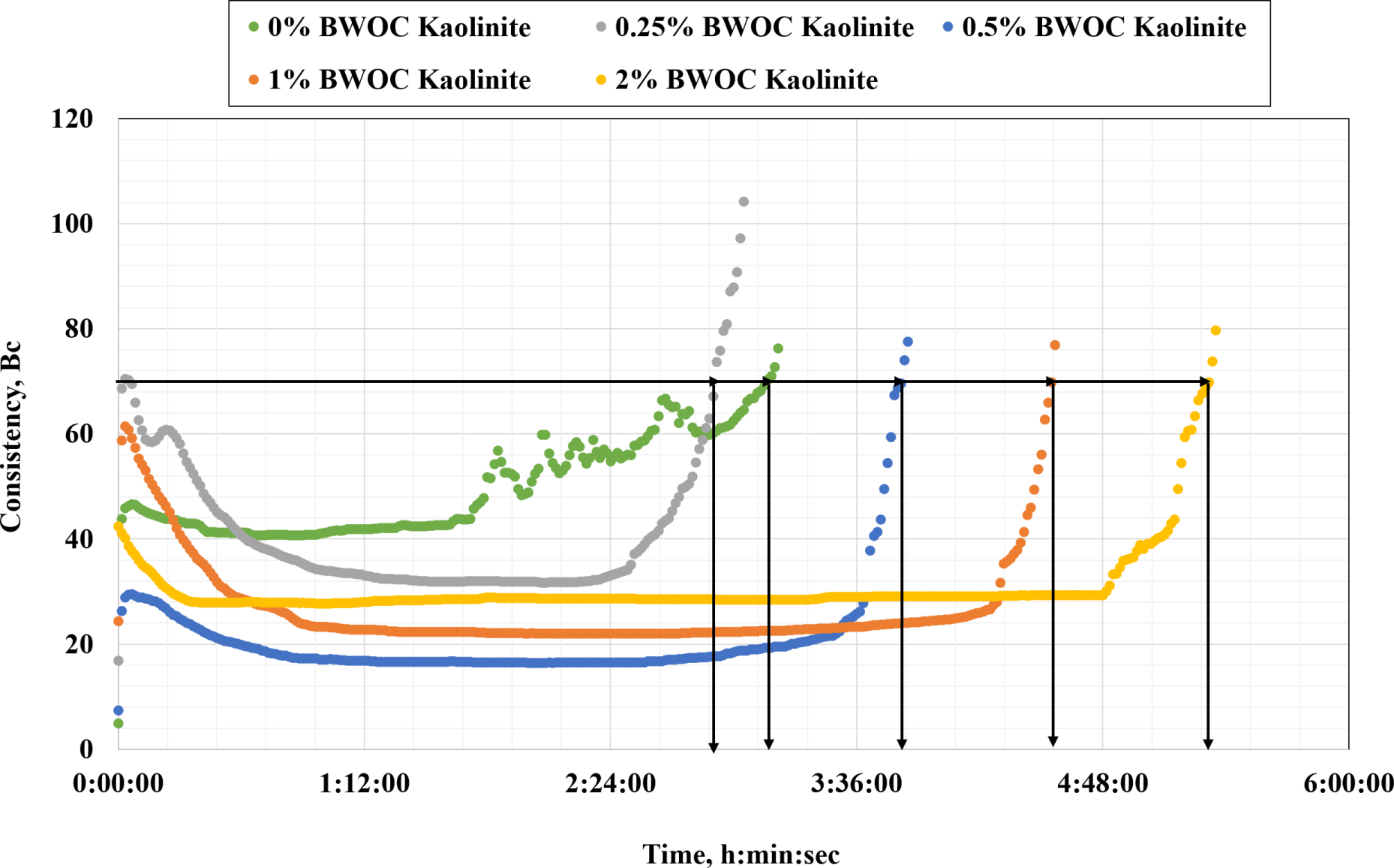
Results of thickening time.

During the placement of cement slurry, it is significant to control its thickening time to avoid early setting and ensure that the slurry remains fluid enough to pump. The use of additives such as kaolinite can help to achieve the desired thickening time of the cement slurry. Therefore, the impact of different dosages of kaolinite on the thickening time was explored, as demonstrated in Fig. 6. The results indicate that the thickening time increased with increasing the amount of kaolinite. At the base cement, the slurry had a thickening time of 190 min at 70 Bc. Using 0.5% BWOC increased the thickening time by 20.5% compared to the base cement to 229 min. Using more amount of kaolinite, such as 1 and 2% BWOC, increased the thickening time by 43.4% and 67.9%, respectively. It can be observed that there was an increase in the thickening time of the cement slurry with increasing concentration of kaolinite which means that the kaolinite can be used as a retarder in oil well cement to delay the time of setting.

This increase in the thickening time for the kaolinite cement slurry can be attributed to the fact that kaolinite is a clay mineral and can adsorb water from the cement slurry, reducing its free water content and slowing down the setting time. While the water adsorption mechanism is important, there are also additional factors at play such as particle interaction, chemical reactions with hydration products, and aluminate phase interactions. For instance, kaolinite particles can physically interfere with the hydration process of cement. The platy structure of kaolinite can create a barrier effect, hindering the contact between cement particles and water. This physical obstruction can slow down the hydration reactions, thereby increasing the thickening time^50^. Kaolinite can react with calcium hydroxide (CH), a primary hydration product of cement, to form additional calcium silicate hydrate (C-S-H) and calcium aluminate hydrate (C-A-H) phases. This pozzolanic reaction consumes CH and can alter the kinetics of cement hydration, potentially leading to a delay in setting time^23^. The high amount of aluminum present in kaolinite can interact with the aluminate phases in cement, potentially forming ettringite or monosulfoaluminate at different rates compared to ordinary Portland cement. This can influence the early hydration reactions and contribute to the retardation effect^51^.

### Results of particle sedimentation through conventional method

The sedimentation of hematite particles in the slurries presents a significant challenge during cementing operations, potentially compromising wellbore durability. This phenomenon occurs as denser particles migrate toward the lower section of the slurry, leading to a concentration of lightweight particles at the top.

**Fig. 7:**
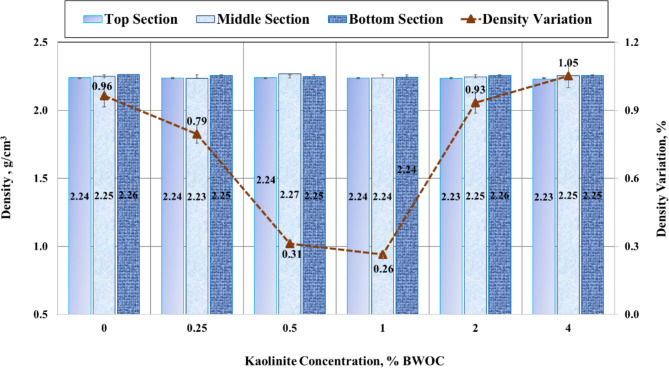
Results of sedimentation test.

Figure 7 illustrates the results of the conventional sedimentation test for each cement sample, detailing the density at the top, middle, and bottom sections. The result reveals that the base case (without kaolinite) exhibits the highest density variation. As kaolinite is introduced, the density variation initially decreases, reaching a minimum before increasing again at higher concentrations. For instance, the base cement sample showed a density variation of 0.96%, with top, middle, and bottom densities of 2.24, 2.25, and 2.26 g/cm^3^, respectively. The addition of 0.25% BWOC kaolinite reduced this variation to 0.79%, yielding densities of 2.24, 2.23, and 2.25 g/cm^3^. A further reduction to 0.31% variation was observed with 0.5% BWOC kaolinite.

The optimal composition was achieved with 1% BWOC kaolinite, resulting in the lowest density deviation of 0.26%, characterized by a uniform density of 2.24 g/cm^3^ across all three sections. However, increasing kaolinite concentration to 2% and 4% BWOC led to increased density variations of 0.93% and 1.05%, respectively. The 4% BWOC sample exhibited the highest variation, with densities of 2.23, 2.25, and 2.25 g/cm^3^ for the top, middle, and bottom sections.

This trend suggests that kaolinite effectively reduces hematite particles settling in oil well cement up to an optimal concentration. The mechanism behind this effect may be attributed to kaolinite’s large surface area and high surface area-to-volume ratio, which enhances its ability to absorb water and other fluids. This property helps maintain consistent water content throughout the cement, thereby mitigating particle settling. Moreover, when incorporated into cement, kaolinite particles can form an interlocking network that impedes the movement of larger cement particles during mixing and placement. This structural arrangement contributes to a more homogeneous distribution of particles throughout the cement matrix. However, the observed increase in density variation at higher kaolinite concentrations (2% and 4% BWOC) suggests that excessive amounts may lead to particle agglomeration and subsequent settling, counteracting the initial stabilizing effect.

One may argue that the change in density between the top and the bottom seems to be abnormal for kaolinite concentrations of 0.25% and 0.5% BWOC. Where the density of the middle part is lower than that of the top (0.25%) and higher than that of the bottom (0.5%). Hence, it should be mentioned that abnormal density variations in the middle or other sections of a sedimentation test can occur due to several factors that influence sedimentation dynamics. For instance, uneven particle distribution may result from inadequate mixing. Additionally, measurement techniques can introduce variability, particularly when dealing with small concentration gradients. Errors in measuring and sampling at different heights can also artificially alter density readings.

However, this variation is not critical and is occasionally observed in reports from cementing service companies. The key criteria for the sedimentation test are as follows: the settled cement drop in the tube should not exceed 5% of the mold length, the average density of the segments should not differ by more than 5% from the measured density, and the density difference between the segment with the highest and lowest density should not exceed 5%. In Fig. 7, the sedimentation test for all concentrations used, including 0.25% and 0.5%, meets these three criteria.

### Results of cement settling through NMR

**Fig. 8:**
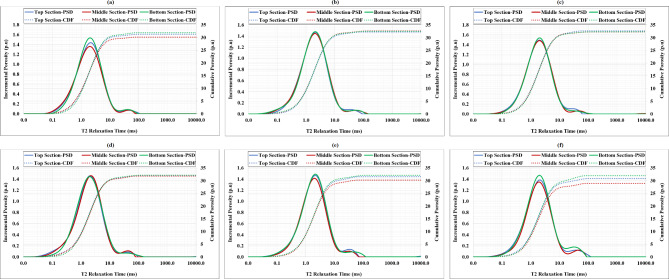
Results of NMR for kaolinite dosages at (**a**) 0%, (**b**) 0.25%, (**c**) 0.5%, (**d**) 1%, (**e**) 2%, and (**f**) 4%.

NMR measurements were employed to corroborate previous findings on sedimentation tests. The probability distribution function (PDF) of cement pores and the cumulative distribution function (CDF) of their porosity were plotted for the upper, middle, and lower parts of the cement specimens, as shown in Fig. 8. The base cement sample ((Fig. 8a)) and the 2% and 4% BWOC kaolinite cement samples (Fig. 8e and f) exhibited significant inconsistencies between the PDF and CDF curves of different sections, reflecting the challenge of maintaining a homogeneous high-density cement while averting sedimentation of particles. In contrast, cement samples containing 0.25%, 0.5%, and 1% BWOC of kaolinite (Fig. 8b and c, and Fig. 8d)) demonstrated remarkably consistent porosity distributions across all sections.

In addition, the porosity measurements of the cement cylinders for the six cement samples were also obtained. The findings showed that the porosity of the 0%, 2%, and 4% BWOC kaolinite cement samples varied significantly between the top, middle, and bottom sections. For example, the base cement sample had porosities of 31.1%, 30.1%, and 31.9% for the upper, middle, and lower sections, respectively as shown in Fig. 8a. The 2% BWOC kaolinite cement had porosities of 31.6%, 30.2%, and 32.1%, for the top, middle and bottom parts, respectively as depicted in Fig. 8e. The 4% BWOC kaolinite cement had porosities of 30.8%, 28.9%, and 32% in the top, middle, and bottom sections, respectively as presented in Fig. 8f. In contrast, the cement samples containing 0.25%, 0.5%, and 1% BWOC of kaolinite had very small or no porosity variation between the top, middle, and bottom sections. The 0.25% BWOC kaolinite cement had porosities of 32.2%, 32.7%, and 32.5%, for the top, middle and bottom sections, respectively as demonstrated in Fig. 8b. The 0.5% BWOC kaolinite cement had porosities of 32.6% (top), 32.3% (in middle), and 32.3% (in the bottom) as revealed in Fig. 8c. The 1% BWOC kaolinite cement had tope, middle, and bottom porosities of 31.9%, 31.8%, and 32.2%, respectively as displayed in Fig. 8d. These results confirm the previous findings that the 0%, 2%, and 4% BWOC kaolinite cement samples had the highest settling issue, while the 0.5 and 1% BWOC kaolinite cement samples had no particle settling.

### Results of petrophysical properties

The porosity and permeability are crucial petrophysical parameters in ensuring wellbore durability throughout its productive lifespan. These properties significantly influence fluid migration behind the casing, a phenomenon that can compromise well integrity^52^. Consequently, a thorough evaluation of these properties in the cement sheath is imperative to avoid costly secondary cementing operations and potential well damage.

**Fig. 9:**
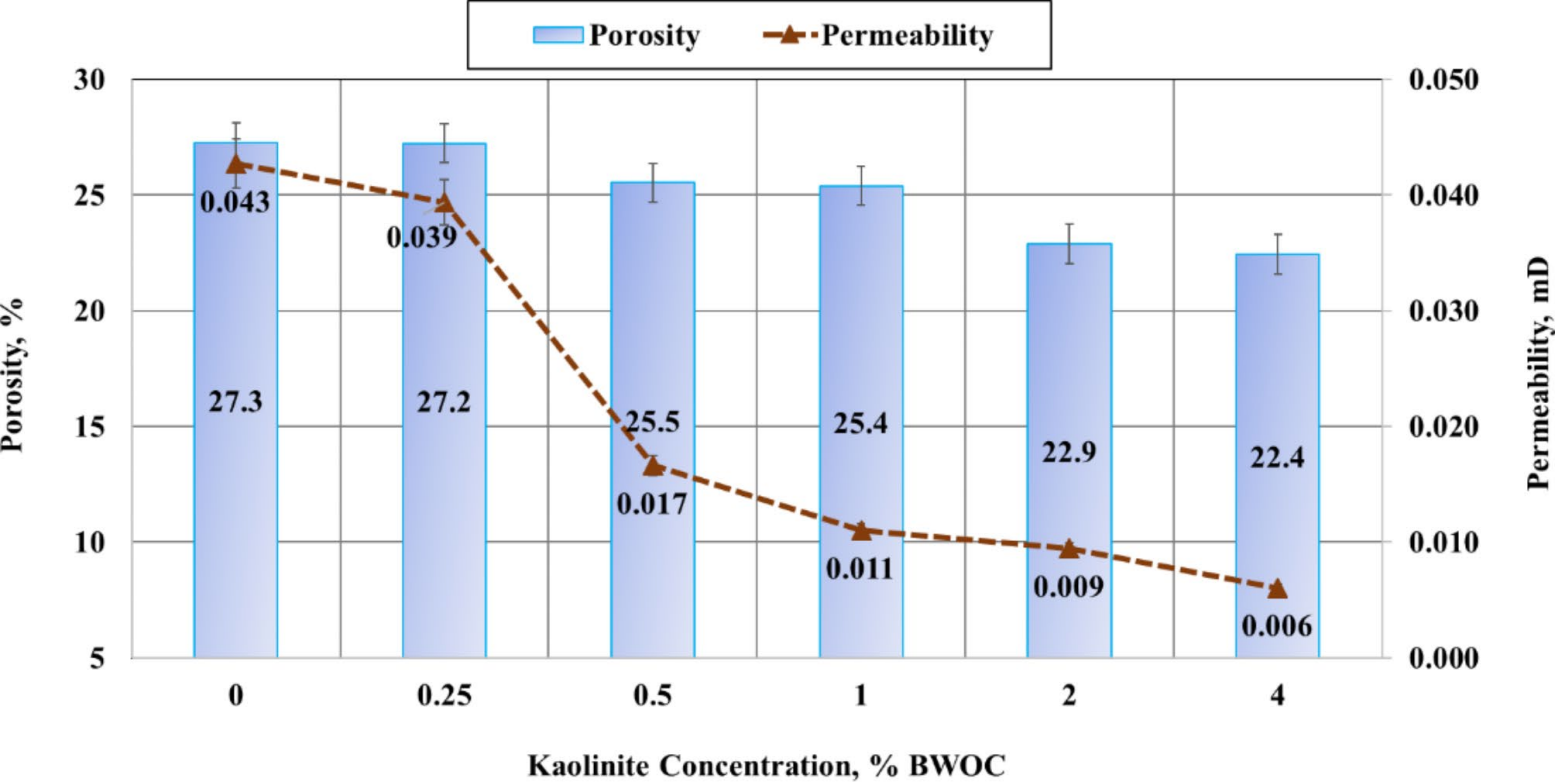
Results of porosity and permeability.

This research examined the influence of several kaolinite dosages on cement porosity and permeability. Figure 9 illustrates a consistent reduction in porosity with increasing kaolinite content. The base sample (0% kaolinite) and the 0.25% BWOC kaolinite sample exhibited similar porosities of 27.3% and 27.2%, respectively. A notable decrease of 7% in porosity was observed when kaolinite concentration increased to 0.5% and 1% BWOC, resulting in a porosity of 25.4%. Further additions of kaolinite (2% and 4% BWOC) led to more significant reductions, with porosities of 22.9% and 22.4%, representing decreases of 16% and 18% compared to the base cement, respectively. While the absolute porosity values differed from those obtained through NMR measurements (Fig. 8), likely due to variations in measurement techniques, the overall trend of decreasing porosity with increasing kaolinite concentration remained consistent across both methods.

Permeability results, also presented in Fig. 9, exhibited a similar trend. The base sample’s initial permeability of 0.043 mD gradually decreased to 0.039 mD with the addition of 0.25% BWOC kaolinite. A sharp reduction of 60.5% in permeability was observed when 0.5% BWOC kaolinite was incorporated. Further increases in kaolinite concentration resulted in progressive permeability reductions, with samples containing 1%, 2%, and 4% BWOC kaolinite showing permeability decreases of 74.4%, 79.1%, and 86.1%, respectively, compared to the base sample.

The observed reductions in both porosity and permeability could be due to the fine particulate nature of kaolinite. These small particles effectively fill the interstitial spaces between cement particles, creating a more compact structure and reducing the available pathways for fluid penetration. This densification of the cement matrix enhances its ability to resist fluid flow, potentially mitigating the risk of inter-zonal fluid migration within the wellbore. These findings have significant implications for wellbore integrity and zonal isolation. The decreased porosity and permeability of kaolinite-modified cement matrices present a more formidable barrier to fluid movement, potentially enhancing the long-term stability and performance of well completions.

### Results of compressive strength

**Fig. 10:**
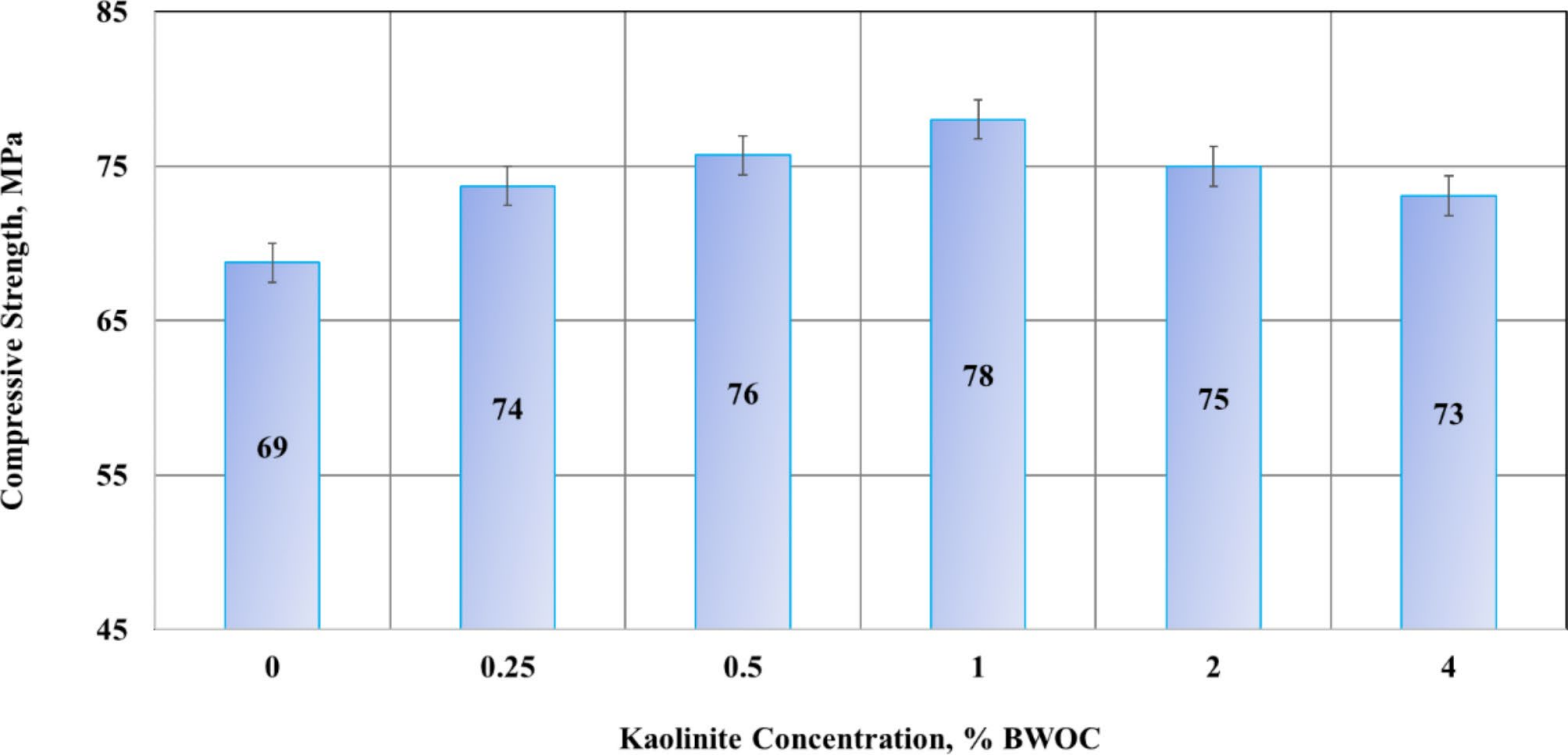
Results of the compressive strength.

The compressive strength of the cement sheath is a critical parameter in the design of cementing operations, as it directly impacts the structural integrity and longevity of the wellbore. This research examined the influence of several kaolinite dosages on the compressive strength of cement samples, with results illustrated in Fig. 10.

The results reveal a strong relationship between kaolinite content and compressive strength. Initially, the addition of kaolinite led to a significant enhancement in compressive strength. The base cement sample, containing no kaolinite, exhibited a compressive strength of 69 MPa. With the introduction of 0.25% BWOC kaolinite, the strength increased by 7.25% to 74 MPa. This upward trend continued with increasing kaolinite content, reaching a peak strength of 78 MPa at 1% BWOC kaolinite which is a 13% improvement over the base sample. However, further increases in kaolinite concentration beyond this optimum point resulted in a decline in compressive strength. Samples containing 2% and 4% BWOC kaolinite showed reduced strengths of 75 MPa and 73 MPa, respectively. This pattern suggests a threshold effect, where excessive kaolinite content begins to compromise rather than enhance cement strength.

Kaolinite’s role in improving strength is multifaceted, involving both improved bonding at the molecular level and densification at the structural level. The strength enhancement due to kaolinite can be understood through both physical and chemical contributions. Kaolinite, being a pozzolanic material, reacts with calcium hydroxide (Ca (OH)_2_), a byproduct of cement hydration, to form an additional calcium silicate hydrate (C-S-H) gel. This chemical interaction improves the mechanical properties by enhancing the bonding strength of the C-S-H, which is primarily responsible for the cement matrix’s strength and durability as explained earlier in Sect. Material characterization for Fig. 2.

Additionally, kaolinite can also contribute to strength improvement by altering the particle composition and potentially increasing density. As a fine-grained material, kaolinite particles can fill voids between cement grains, decreasing permeability and porosity and leading to a denser microstructure. A denser microstructure generally correlates with higher strength as explained earlier in Sect. Material characterization for Table 1. This physical effect is known as the filler effect or particle packing effect.

The subsequent decline in strength at higher kaolinite concentrations (2% and 4% BWOC) may be due to particle agglomeration. Excessive kaolinite content could lead to the formation of clusters that disrupt the optimal cement microstructure, potentially creating weak points or inhomogeneities within the matrix. These findings have significant implications for wellbore integrity. Enhanced compressive strength, as observed with optimal kaolinite additions, translates to improved resistance against collapse and cracking of the cement sheath. This increased structural integrity is crucial for protecting the casing and maintaining wellbore stability over extended periods.

## Conclusions and recommendations

This study evaluates the efficacy of kaolinite as an additive in oil well cement formulations. Comprehensive characterization of kaolinite particles was conducted using particle size distribution, quantitative evaluation of materials scanning (QEMscan), and X-ray fluorescence (XRF). Through systematic examination of various kaolinite concentrations, an optimal dosage of 1% by weight of cement (BWOC) was identified. The incorporation of kaolinite particles into oil well cement yielded significant improvements across multiple performance parameters:

- Rheological properties: kaolinite addition resulted in an 8.4% reduction in plastic viscosity compared to the base cement, while simultaneously increasing yield point and gel strength by 19.4% and 30%, respectively.
- Setting time control: kaolinite demonstrated retarding properties, with increasing concentrations correlating to extended thickening times of the cement slurry.
- Particle settling mitigation: in heavy-weight cement systems utilizing hematite, kaolinite inclusion minimized particle settling, reducing density variation to 0.26% compared to 1% in the base sample.
- Homogeneity confirmation: nuclear magnetic resonance (NMR) analysis corroborated kaolinite’s effectiveness in mitigating hematite particle settling, evidenced by uniform porosity distributions along the length of kaolinite-modified cement samples.
- Pore structure refinement: increasing kaolinite concentrations led to reductions in both permeability and porosity, indicating effective pore-filling capabilities.
- Strength enhancement: the incorporation of kaolinite particles resulted in a 13% increase in compressive strength compared to the base cement formulation.

The following points are recommended for the future work:

- Considering the long-term effects of the kaolinite on the cement properties by studying the effect of several curing times such as 6 h, 12 h, 24 h, 48 h, 3 days, 7 days, 14 days, and 28 days on the properties of kaolinite-based cement samples.
- Considering the exposure to several temperatures and investigating how temperature changes influence kaolinite performance.
- Conducting analytical techniques such as X-ray diffraction (XRD) to detect phase changes and Fourier-transform infrared spectroscopy (FTIR) to assess the chemical interactions between kaolinite and the hydration products.

## Data Availability

“No external data was used for this research. All the generated experimental data are included in this manuscript. The datasets used and/or analysed during the current study available from the corresponding author on reasonable request.”
